# Effect of Aqueous Extracts of Orange Peel Biochar on Seed Germination and Early Seedling Growth of Durum Wheat (*Triticum durum* Desf.) and Common Buckwheat (*Fagopyrum esculentum* Moench.)

**DOI:** 10.3390/plants15091292

**Published:** 2026-04-22

**Authors:** Barbora Tunklová, Jan Velebil, Jan Malaťák, Monika Aniszewska

**Affiliations:** 1 Czech Agrifood Research Center, Drnovská 507/73, 161 00 Prague, Czech Republic; 2 Faculty of Engineering, Czech University of Life Sciences Prague, Kamýcká 129, 165 00 Prague, Czech Republic; velebil@tf.czu.cz (J.V.); malatak@tf.czu.cz (J.M.); 3 Department of Biosystems Engineering, Institute of Mechanical Engineering, Warsaw University of Life Sciences, Nowoursynowska 164, 02-787 Warsaw, Poland; monika_aniszewska@sggw.edu.pl

**Keywords:** lignocellulosic waste, phytotoxicity, secondary metabolism, pyrolysis, plant stress response

## Abstract

This study investigated the effects of aqueous extracts of orange peel–derived biochar on seed germination and early seedling growth in durum wheat (*Triticum durum* Desf.) and common buckwheat (*Fagopyrum esculentum* Moench.). Biochar was produced by pyrolysis of orange peel at temperatures ranging from 250 to 550 °C. Germination assays were conducted under controlled laboratory conditions, and seedling growth parameters were evaluated after six days of cultivation. Untreated orange peel completely inhibited seed germination (0 %) in both species, while biochar produced at 250 °C significantly reduced germination (e.g., the germination index decreased from 54.21 % in the control to 47.2 % in *T. durum*). In contrast, biochar produced at 350 °C increased germination to >96 % in T. durum and 100 % in *F. esculentum*, accompanied by enhanced seedling vigor and biomass production. Chemical analyses revealed a pronounced decrease in total phenolic content (from 53.84 to 0.57 mg GAE g^−1^ DW) and flavonoids (from 90.05 to 1.34 mg QE g^−1^ DW) with increasing pyrolysis temperature, along with a reduction in antioxidant activity. Common buckwheat exhibited consistently higher tolerance to biochar extracts than durum wheat across all treatments. Overall, the results demonstrate that pyrolysis temperature is a key factor governing the transition from phytotoxic to biostimulatory effects, with optimal performance observed at approximately 350 °C.

## 1. Introduction

Global citrus production exceeds 110 million tonnes annually, with oranges accounting for approximately 60 % of the total output [1]. A substantial proportion of citrus fruits is processed into juice, generating large quantities of by-products such as peels, segment membranes, and pulp. These residues, collectively referred to as citrus waste, represent an important biomass stream with considerable potential for valorization [2,3]. Common management strategies for citrus waste include composting, anaerobic digestion, incineration, gasification, and pyrolysis [4,5]. Nevertheless, a significant fraction of citrus waste is still disposed of in landfills, resulting in the loss of valuable organic matter and nutrients and contributing to environmental pollution [6].

Orange peel constitutes a major fraction of citrus waste and is rich in carbohydrates, cellulose, hemicellulose, pectin, and a wide range of secondary metabolites, including phenolic compounds, flavonoids, and essential oils [7,8,9]. Among these compounds, limonene is particularly abundant and has been identified as a potent phytotoxic agent capable of inhibiting seed germination and early seedling development [10,11,12]. Consequently, the direct application of untreated orange peel or its aqueous extracts may negatively affect crop establishment and early growth stages.

The conversion of citrus waste into biochar via pyrolysis has emerged as a promising strategy for biomass valorization and environmentally sustainable waste management [13,14,15]. Biochar is defined as a porous carbonaceous material produced by the thermal decomposition of biomass under oxygen-limited conditions and intended for applications other than energy production [16]. Biochar can be produced over a broad temperature range, typically between 200 and 700 °C, and its physicochemical properties strongly depend on feedstock type and pyrolysis parameters, particularly temperature [17,18,19].

Increasing pyrolysis temperature generally results in higher carbon content and increased aromaticity, accompanied by a decrease in hydrogen and oxygen contents, thereby influencing biochar stability and reactivity [20,21]. Biochar typically contains high proportions of carbon (55–95 % wt.) and varying amounts of biogenic elements such as nitrogen, phosphorus, potassium, calcium, magnesium, and trace metals, depending on the original biomass and processing conditions [18,22]. Owing to its porous structure and large specific surface area, biochar exhibits strong sorption capacity for inorganic ions and organic compounds, which can influence nutrient retention and contaminant mobility in soils [23,24,25].

In addition to these properties, biochar may also contain potentially hazardous constituents originating from the feedstock or formed during thermal conversion. Trace elements, including heavy metals, may be present in the original biomass and can become relatively enriched during pyrolysis due to the progressive loss of organic matter and concentration of the inorganic fraction [10,26]. The final concentration of these elements depends on the feedstock origin, environmental conditions, and possible contamination during cultivation and processing. Furthermore, citrus peels may contain residues of post-harvest treatments, such as fungicides and other chemical preservatives applied to prolong storage stability. These compounds may persist in the biomass and potentially influence the chemical composition and biological activity of the resulting biochar or its aqueous extracts [27,28,29,30]. Therefore, consideration of feedstock quality and potential contaminants is essential when evaluating citrus-derived biochar for agricultural use.

Numerous studies have demonstrated that biochar application can improve soil structure, increase water-holding capacity, enhance nutrient retention, and stimulate microbial activity, thereby supporting plant growth and soil fertility [31,32,33,34]. Moreover, biochar has been recognized as an effective tool for climate change mitigation through long-term carbon sequestration and the partial substitution of fossil-based materials [35,36].

Despite these potential benefits, biochar may also exert negative effects on plants, particularly during seed germination and early seedling development. These adverse effects have been attributed to the presence of water-soluble phenolic compounds, volatile organic compounds, polycyclic aromatic hydrocarbons (PAHs), polychlorinated biphenyls (PCBs), excessive alkalinity, or osmotic stress caused by elevated concentrations of soluble salts [27,28,29,30]. Consequently, standardized phytotoxicity testing prior to agricultural application is strongly recommended. According to OECD guidelines, seeds of garden cress (*Lepidium sativum* L.), lettuce (*Lactuca sativa* L.), and wheat (*Triticum aestivum* L.) are commonly used for phytotoxicity assessment due to their rapid germination and high sensitivity to toxic substances.

To mitigate phytotoxic effects, several strategies have been proposed, including washing biochar with water or organic solvents to remove soluble toxic compounds and excess alkalinity [28,37,38]. However, such treatments may also result in the loss of soluble nutrients (e.g., Ca, Mg, K, N, and P) and induce changes in surface morphology and chemical reactivity, potentially reducing the agronomic effectiveness of biochar [39].

An alternative and widely applied approach for biochar evaluation involves the use of aqueous biochar extracts, which provide a sensitive and reproducible tool for assessing the biological effects of water-soluble biochar constituents on seed germination and early seedling growth [24,29]. Depending on biochar properties and plant species, biochar extracts have been reported to exert both stimulatory and inhibitory effects on early plant development [40].

Despite increasing interest in biochar derived from agricultural and food-processing residues, data on the phytotoxicity and biostimulatory potential of biochar produced from citrus waste, particularly orange peel, remain scarce. Moreover, the influence of pyrolysis temperature on the biological effects of aqueous extracts of orange peel biochar has not yet been systematically investigated.

Therefore, the present study focuses on biochar produced from orange peel and evaluates the effects of its aqueous extracts on seed germination and early seedling growth of durum wheat (*Triticum durum* Desf.) and common buckwheat (*Fagopyrum esculentum* Moench.). These species were selected to represent a cereal and a pseudocereal crop with differing physiological sensitivities. The objectives of this study were to (i) assess the phytotoxic or stimulatory effects of aqueous extracts of orange peel biochar, (ii) compare species-specific responses, and (iii) identify an optimal pyrolysis temperature that minimizes phytotoxicity while supporting early plant development.

## 2. Results

### 2.1. Elemental Composition, pH, and Electrical Conductivity of Orange Peel Biochar

The concentrations of potentially toxic elements (Cr, Ni, Cu, Zn, As, Cd, Pb, and Hg) measured in untreated orange peel and orange peel biochars produced at different pyrolysis temperatures are presented in Table 1. Thermal treatment of biomass is known to result in the relative enrichment of inorganic components due to progressive organic matter degradation, which was reflected in the observed increase in most elemental concentrations with increasing pyrolysis temperature. Chromium concentrations increased from 2.51 mg·kg^−1^ in untreated orange peel to 7.41 mg·kg^−1^ in biochar produced at 550 °C, while nickel concentrations increased from 3.95 to 9.62 mg·kg^−1^ across the same temperature range (Table 1). Copper concentrations increased with pyrolysis temperature, reaching a maximum of 8.22 mg·kg^−1^ at 450 °C, whereas zinc showed pronounced enrichment following thermal conversion, with concentrations increasing from 9.79 mg·kg^−1^ in untreated peel to values between 17.33 and 26.18 mg·kg^−1^ in biochars (Table 1). Arsenic concentrations increased to a maximum of 0.94 mg·kg^−1^ at 350 °C and slightly decreased at higher temperatures, while cadmium concentrations remained below the detection limit in all samples. Lead concentrations increased moderately with temperature, and mercury concentrations remained very low across all treatments. The pH and electrical conductivity (EC) of aqueous extracts are summarized in Table 2. Untreated orange peel exhibited acidic pH (4.26), whereas pyrolysis led to a pronounced increase in alkalinity. Near-neutral pH values were observed for biochars produced at 250–300 °C, while strongly alkaline conditions (pH > 9.5) were recorded at higher temperatures. Electrical conductivity showed substantial variation among samples. The lowest EC was recorded for biochar produced at 300 °C (771.8 μS·cm^−1^), whereas the highest EC values occurred at 450 °C and 550 °C (2259 and 3080 μS·cm^−1^, respectively).

### 2.2. Effect of Pyrolysis Temperature on Secondary Metabolites

Detailed total phenolics (TPC), flavonoids (TFC), and total antioxidant activity (TAA) can be seen in Table 3. Untreated orange peel exhibited extremely high concentrations of flavonoids (90.05 ± 3.40 mg QE g^−1^ DW), phenolic compounds (53.84 ± 0.69 mg GAE g^−1^ DW), and total antioxidant activity (27.58 ± 0.05 mg AAE g^−1^ DW).

Pyrolysis at 250 °C led to a substantial reduction in the content of secondary metabolites; however, flavonoid (8.07 ± 0.04 mg QE g^−1^ DW) and phenolic contents (4.77 ± 0.08 mg GAE g^−1^ DW) remained relatively high. This residual concentration of phytotoxic compounds explains the persistence of inhibitory effects observed for biochar extracts produced at this temperature.

With increasing pyrolysis temperature, a pronounced decline in all measured parameters was observed. The most significant reduction occurred at 350 °C, where flavonoid and phenolic contents decreased to 1.34 ± 0.02 mg QE g^−1^ DW and 0.57 ± 0.01 mg GAE g^−1^ DW, respectively. Further increases in temperature to 450 °C and 550 °C resulted in only minor additional reductions. These results indicate that a pyrolysis temperature of approximately 350 °C represents a critical threshold at which phytotoxic secondary metabolites are effectively degraded while some potentially beneficial, biologically active compounds may still remain.

### 2.3. Effects on Seed Germination and Early Seedling Growth

The effects of aqueous extracts of untreated orange peel and orange peel–derived biochars on seed germination and early seedling growth of *Triticum durum* and *Fagopyrum esculentum* are summarized in Table 4 and Table 5. Aqueous extracts of untreated orange peel completely inhibited germination in both species, as no seed emergence or seedling development was observed. These results confirm that untreated citrus biomass is unsuitable for direct agronomic application due to its strong inhibitory potential [41]. In contrast, aqueous extracts of orange peel biochars exhibited a clear dependence on pyrolysis temperature, with both inhibitory and stimulatory effects observed. Biochar produced at 250 °C reduced germination performance and early seedling growth, particularly in *Triticum durum*. In this species, the germination index decreased from 54.21 % in the control to 47.25 %, and seedling vigor index I declined from 121.26 to 78.48 mm. Root elongation on the third day was markedly reduced relative to the control, indicating delayed early development. In *Fagopyrum esculentum*, the inhibitory effect at 250 °C was less pronounced, with final germination reaching 100 % and seedling vigor index I exceeding the control value. This species-specific response has been reported previously and is often linked to differences in seed coat permeability, detoxification capacity, and metabolic plasticity, which influence tolerance to biochar-derived compounds [30,42]. At intermediate pyrolysis temperatures (300–350 °C), aqueous biochar extracts markedly stimulated germination and early seedling development in both species. Germination rates exceeded 96 % in *Triticum durum* and reached 100 % in *Fagopyrum esculentum*. Seedling vigor indices increased substantially, with particularly pronounced enhancement at 350 °C, where seedling vigor index II and III reached 4.52 mg and 0.34 mg in wheat and 3.07 mg and 0.24 mg in buckwheat, respectively. Enhanced root and shoot elongation and increased biomass accumulation were also observed. The temperature-dependent stimulation observed in the present study closely resembles responses reported for non-thermal physicochemical treatments, particularly non-thermal atmospheric pressure plasma. At higher pyrolysis temperatures (450–550 °C), final germination in both species remained comparable to the control (≈98–100 %), indicating the absence of phytotoxic effects. Seedling vigor index I further increased, reaching 137.04–144.03 mm in *Triticum durum* and 149.34–157.51 mm in *Fagopyrum esculentum*. However, biomass-related parameters showed smaller increases or plateaued relative to intermediate temperatures, and root-to-shoot ratios indicated reduced shoot stimulation. Overall, the results demonstrate that aqueous extracts of orange peel biochars exert strong, temperature-dependent effects on seed germination and early seedling growth, with inhibition at low pyrolysis temperature, maximum stimulation at intermediate temperatures, and reduced stimulatory potential at higher temperatures. When viewed alongside findings from non-thermal atmospheric pressure plasma treatments, these results highlight that both thermal and non-thermal modification strategies can effectively enhance early plant development when applied in an optimal dose.

### 2.4. Species-Specific Responses

Throughout the experiment, common buckwheat (*Fagopyrum esculentum*) exhibited higher tolerance to orange peel biochar extracts than durum wheat (*Triticum durum*). Buckwheat consistently maintained higher germination percentages, longer root systems, and greater seedling vitality indices across most treatments.

The higher tolerance of buckwheat suggests that this species may be more suitable for environments where biochar application carries a potential risk of residual phytotoxicity, particularly when biochars are produced at lower pyrolysis temperatures.

## 3. Discussion

The results of this study clearly demonstrate that aqueous extracts of orange peel–derived biochar exert pronounced and strongly pyrolysis-temperature-dependent effects on seed germination and early seedling growth of *Triticum durum* and *Fagopyrum esculentum*. The complete inhibition of germination observed for untreated orange peel is fully consistent with numerous reports describing the strong phytotoxicity of citrus residues. This effect has been primarily attributed to the high abundance of monoterpenes, particularly limonene, together with phenolic compounds and flavonoids that are readily released into aqueous extracts [38,43]. These compounds are known to disrupt membrane integrity, inhibit mitochondrial respiration, and interfere with enzymatic pathways involved in energy metabolism during germination, ultimately suppressing radicle emergence and seedling establishment. Low-temperature pyrolysis (250 °C) substantially reduced, but did not fully eliminate, the phytotoxic potential of orange peel. Residual concentrations of phenolics and flavonoids detected in biochar extracts produced at this temperature provide a plausible explanation for the inhibitory effects observed, particularly in *T. durum*. Similar inhibitory responses to low-temperature biochars have been widely reported and are commonly associated with the persistence of mobile organic compounds and partially decomposed oxygen-containing functional groups that remain bioavailable in aqueous extracts [24,44]. In addition to organic phytotoxins, elevated electrical conductivity measured in low-temperature biochar extracts may contribute to osmotic stress, which has been shown to adversely affect germination kinetics and early seedling growth [31].

In contrast, biochars produced at intermediate pyrolysis temperatures (300–350 °C) exhibited a pronounced stimulatory effect on seed germination and early seedling development in both test species. At these temperatures, concentrations of phytotoxic secondary metabolites declined sharply, with particularly pronounced reductions observed at 350 °C. Previous studies have identified similar temperature thresholds for the thermal degradation of phenolic compounds and other labile phytotoxins during biomass pyrolysis [14,45,46]. These findings indicate that moderate pyrolysis temperatures are sufficient to suppress inhibitory effects while preserving biochar fractions capable of exerting positive biological activity.

The enhanced germination percentages, increased root elongation, and higher biomass accumulation observed at 350 °C are consistent with earlier reports demonstrating the biostimulatory potential of biochar water extracts on early plant development [39,47]. Such stimulation has been linked to several non-exclusive mechanisms, including increased availability of mineral nutrients, the presence of low-molecular-weight organic compounds [23,32] acting as signaling molecules, and modulation of hormonal and redox balance during early developmental stages [17,18]. Although aqueous extracts cannot fully replicate soil–biochar–plant interactions, they represent a sensitive and reproducible approach for isolating the biological effects of water-soluble biochar constituents. At higher pyrolysis temperatures (450–550 °C), phytotoxic effects were completely eliminated, as evidenced by germination rates comparable to the control. However, the stimulatory effects on biomass accumulation and seedling vigor indices were reduced relative to intermediate temperatures. This attenuation of biological activity has been attributed to increased aromaticity, condensation of carbon structures, and reduced polarity of organic compounds in high-temperature biochars, which collectively limit their solubility and bioavailability in aqueous extracts [15,46]. Moreover, the strongly alkaline pH and elevated electrical conductivity observed at higher temperatures may influence nutrient uptake and biomass allocation patterns during early growth, as reflected by changes in root-to-shoot ratios [48]. Beyond organic compounds, pyrolysis also induced systematic changes in the inorganic composition of orange peel biochar. Similar temperature-dependent enrichment trends for Cr and Ni have been reported for biochars derived from crop residues and food-processing waste streams, reflecting progressive concentration of inorganic constituents during thermal degradation of organic matter [10,26]. The enrichment of Zn and Cu with increasing pyrolysis temperature has been attributed to their low volatility and strong association with mineral ash fractions, which become increasingly concentrated as pyrolysis proceeds [49]. Such element-specific behavior reflects differences in elemental volatility, chemical speciation, and bonding strength within the biomass matrix [23]. Importantly, despite this relative enrichment, the concentrations of all monitored potentially toxic elements remained well below the threshold limits defined by the European Biochar Certificate, indicating no immediate environmental or agronomic risk [11]. The observed increase in pH with rising pyrolysis temperature is a well-established phenomenon and is primarily associated with the accumulation of alkaline ash constituents, including carbonates and oxides of alkali and alkaline earth metals [12,50]. Elevated electrical conductivity at higher pyrolysis temperatures reflects increased concentrations of water-soluble salts, which have been shown to influence osmotic conditions in germination assays and may partially explain changes in seedling growth patterns at these temperatures [51,52]. The overall temperature-dependent response pattern observed in this study—characterized by strong phytotoxicity at low temperatures, maximum stimulation at intermediate temperatures, and reduced stimulation at high temperatures—closely aligns with trends reported for biochars derived from a wide range of feedstocks and production conditions [53]. Interestingly, analogous threshold-dependent effects have also been described for non-thermal atmospheric pressure plasma treatments, where optimized exposure enhances seed germination and early seedling growth, while insufficient or excessive exposure results in reduced or inhibitory responses [54]. In that study, growth stimulation was attributed to plasma-induced surface activation, enhanced wettability, and the generation of reactive species that modulate early metabolic processes. Despite fundamentally different mechanisms, both thermal and non-thermal treatments appear to share common biological response thresholds separating inhibitory and stimulatory effects. Species-specific responses were evident throughout the experiment. *F*. *esculentum* consistently exhibited higher tolerance to orange peel biochar extracts than *T. durum*, particularly under low-temperature biochar treatments. Such interspecific variability has been widely reported and is often attributed to differences in seed coat permeability, antioxidant capacity, metabolic plasticity, and detoxification mechanisms among plant species [30,42]. The higher tolerance of buckwheat suggests that this species may be more resilient to residual phytotoxicity associated with suboptimally produced biochars and may therefore represent a suitable model crop for environments where biochar quality cannot be fully controlled. Overall, the results confirm that pyrolysis temperature is a critical determinant of the phytotoxic and biostimulatory properties of orange peel biochar. When properly optimized, thermal conversion can transform citrus waste from a strongly inhibitory material into a biologically active amendment capable of enhancing early plant development. These findings underscore the importance of standardized phytotoxicity testing, careful optimization of biochar production parameters, and species-specific evaluation prior to the agricultural application of biochar derived from citrus residues.

## 4. Materials and Methods

### 4.1. Plant Material

**Figure 1:**
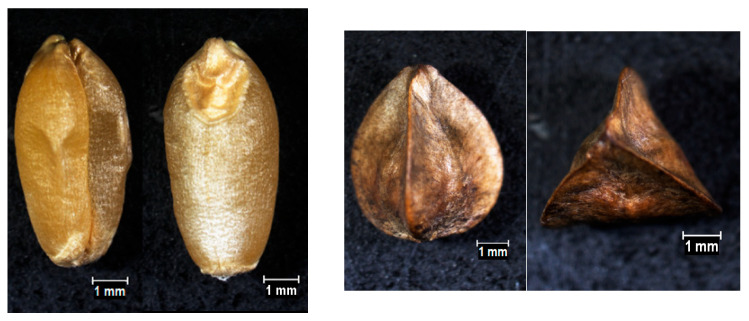
Durum wheat grains and common buckwheat achenes were examined under a Nikon SMZ 800 stereomicroscope (Nikon, Tokyo, Japan), and images were captured using NIS-Elements AR 4.30 software.

Cereal grains of durum wheat (*Triticum durum* Desf.) and pseudocereal achenes of common buckwheat (*Fagopyrum esculentum* Moench.) were purchased from commercial suppliers in the Czech Republic (Figure 1). The thousand-grain/achene weights were 52 g for durum wheat and 24 g for buckwheat. Seed germination ranged from 80 to 95 % for durum wheat and 80–85 % for buckwheat. Only fully mature, undamaged, and untreated (unpeeled) seeds were selected for the experiment, ensuring that the surface of the fruits was intact. For each treatment group (different treatments with orange peel extract) as well as for the control samples, 150 seeds per plant species were used.

### 4.2. Preparation of Biochar Samples

For the preparation of the biochar samples, orange peel was sourced from a juice processing facility. The peel was first dried in ambient air; subsequently, it was dried slowly in a laboratory oven at 80 °C and ground to a particle size below 1 mm. The biochar samples were prepared in a programmable furnace (LECO TGA701, LECO Instruments, St. Joseph, MI, USA), where an inert atmosphere was guaranteed by N_2_ flushing during the pyrolysis phase. The temperature program included first drying at 105 °C to constant weight and then a temperature increase at 20 K min^−1^ to the target temperature, at which it was held for 30 min.

### 4.3. Preparation of Aqueous Extracts

Aqueous extracts were prepared from raw pyrolyzed material and orange peel biochar according to a previously described method [55,56]. Briefly, 10 g of the original material or biochar was placed into an Erlenmeyer flask, and distilled water was added in an amount corresponding to ten times the dry matter weight of the sample. The flasks containing the biochar–water mixture were placed on a horizontal shaker and agitated for 2 h at room temperature. Subsequently, the suspension was filtered, and the resulting filtrate was used for further analyses and germination experiments. Based on the applied methodology, the final extract concentration corresponded to a 10 % (*w*/*v*) aqueous solution, which was consistently used in all experiments.

### 4.4. Seed Germination and Seedling Growth Bioassay

Seed germination tests were conducted in glass Petri dishes (120 mm in diameter) lined with filter paper (Papírna Perštejn KA0/110, Perštejn, Czech Republic). Thirty seeds and 7 mL of the respective aqueous extract were applied to each Petri dish. Distilled water served as the control treatment. Each treatment and control variant was replicated five times (five Petri dishes per treatment), resulting in a total of 150 seeds per treatment.

All Petri dishes were placed in a growth chamber under dark conditions at a constant temperature of approximately 25 °C. The cultivation period lasted 6 days. A seed was considered germinated when the radicle length exceeded 2 mm.

The number of germinated seeds, shoot length, and root length were recorded on the 3rd and 6th day of the experiment. Fresh weight (FW) of shoots and roots was measured on day 6. Subsequently, shoots and roots were dried separately at 105 °C for 3 days, and dry weight (DW) was determined.

Seed germination on the 3rd and 6th day (G_3_, G_6_) was calculated as the percentage ratio between the number of germinated seeds and the total number of seeds used in the test. Germination rate (GR) and germination index (GI) were calculated according to the methodology described in [55]. Seedling length (L) was determined as the sum of root length (LR_6_) and shoot length (LS_6_). Fresh (WF) and dry weight (WD) of seedlings were calculated as the sum of shoot and root weights.

Root-to-shoot ratios were calculated based on length (R/S_L), fresh weight (R/S_FW), and dry weight (R/S_DW) measured on day 6. Three seedling vigor indices were determined: seedling vigor index I (SVI_I), defined as seed germination × seedling length; seedling vigor index II (SVI_II), seed germination × seedling fresh weight; and seedling vigor index III (SVI_III), seed germination × seedling dry weight. A detailed description of the evaluated parameters is provided in [55,57,58].

### 4.5. Preparation of Methanolic Extracts

Methanolic extracts were prepared in accordance with the method described by Tunklová et al. [59] for the determination of total phenolic content, total flavonoid content, total antioxidant activity, and tannin content. Briefly, 0.2 g of sample was extracted with 5 mL of 70 % methanol. The mixture was subjected to ultrasonic extraction at 70 °C for 45 min. Subsequently, the samples were centrifuged at 2350× *g* for 15 min. The supernatant was collected in a 10 mL volumetric flask. The extraction procedure was repeated once, and the combined extracts were adjusted to a final volume of 10 mL. Each methanolic extract was prepared in triplicate.

### 4.6. Determination of Total Phenolic Content, Total Flavonoid Content, and Total Antioxidant Activity

Total phenolic content (TPC) was determined using the Folin–Ciocalteu method (Sigma-Aldrich, Saint Louis, MO, USA) according to Singleton and Rossi [60] and Tsantili et al. [61]. Results are expressed as gallic acid equivalents (GAE, mg·g^−1^ DW).

Total flavonoid content (TFC) was determined by a colorimetric method using aluminum chloride according to Chang et al. [62]. Results are expressed as quercetin equivalents (QE, mg·g^−1^ DW).

Total antioxidant activity (TAA) was assessed using the phosphomolybdenum method as described by Subhasree et al. [63] and expressed as ascorbic acid equivalents (AAE, mg·g^−1^ DW).

### 4.7. pH, Electrical Conductivity, and Elemental Analysis

The pH of the prepared orange peel aqueous extracts was measured using a pH meter (Mettler Toledo MP220, Mettler Toledo, Columbus, OH, USA). Electrical conductivity was determined using a conductometer equipped with a conductivity electrode.

For the determination of total elemental contents in the orange peel, 0.2 g of dry sample was digested using 4 mL of HNO_3_ and 2 mL of H_2_O_2_ (Rotipuran^®^, Carl Roth, Karlsruhe, Germany). Quality assurance was ensured by parallel analysis of certified reference materials (CRM; Metranal 31, Analytika s.r.o., Prague, Czech Republic; Peach Leaves SRM 1547, NIST) and procedural blanks. High-purity water (≥18.2 MΩ·cm; Milli-Q purification system, Millipore SAS, Molsheim, France) was used for all dilutions and sample preparations. For elemental analysis of biochars, 0.5 g of dry biochar was weighed into a 35 mL quartz digestion vessel. Subsequently, 1.5 mL of HNO_3_ and 4.5 mL of HCl (both Analpure^®^, Analytika s.r.o., Prague, Czech Republic) were added along with a Teflon-coated stir bar. The vessels were sealed and subjected to microwave-assisted digestion (Discover SP-D, CEM Corp., Stallings, NC, USA) at 180 °C for 18 min. The concentrations of As, Cd, Cr, Cu, Ni, Pb, Hg and Zn were determined in diluted digests using inductively coupled plasma quadrupole mass spectrometry (ICP-MS; Agilent 7700x, Agilent Technologies Inc., Santa Clara, CA, USA) operated in helium mode to reduce polyatomic interferences.

Each experimental variant was performed in three repetitions, whereas germination tests were conducted in five biological replicates.

### 4.8. Data Analyses

All measured data were subjected to analysis of variance (ANOVA) using STATISTICA software (Statistica 13, StatSoft Inc., Tulsa, OK, USA). One-way ANOVA was applied to evaluate the effect of individual treatments on the observed parameters. Differences among means were further assessed using Tukey’s HSD test for multiple comparisons at a significance level of *p* ≤ 0.05. Significant differences between treatments are indicated by different letters.

## 5. Conclusions

This study demonstrated that orange peel–derived biochar can exert either strong phytotoxic or pronounced biostimulatory effects on seed germination and early seedling growth, depending primarily on the applied pyrolysis temperature. Untreated orange peel and biochar produced at low temperature (250 °C) exhibited marked phytotoxicity, resulting in complete or substantial inhibition of germination and early growth. These negative effects were closely associated with high concentrations of water-soluble secondary metabolites, including phenolic compounds, flavonoids, and limonene, which are known to interfere with key physiological processes during germination.

In contrast, biochar produced at intermediate pyrolysis temperatures, particularly at 350 °C, showed a clear and consistent stimulatory effect on germination, root and shoot elongation, biomass accumulation, and seedling vitality in both tested species. This response coincided with a sharp decline in phytotoxic secondary metabolites while retaining sufficient amounts of water-soluble compounds and nutrients capable of promoting early plant development. Biochars produced at higher temperatures (450–550 °C) no longer exhibited phytotoxic effects; however, their biostimulatory potential was reduced, likely due to increased aromaticity, reduced polarity, and limited release of biologically active compounds into aqueous extracts.

Common buckwheat consistently exhibited higher tolerance to orange peel biochar extracts than durum wheat, highlighting the importance of species-specific sensitivity in phytotoxicity and biostimulation assessments. Importantly, despite temperature-dependent enrichment of inorganic elements during pyrolysis, all measured concentrations of potentially toxic elements remained well below the limits defined by the European Biochar Certificate, confirming the suitability of orange peel as a safe feedstock for biochar production under controlled conditions.

Overall, the results identify pyrolysis temperature as a critical factor governing the transition of orange peel biochar from a phytotoxic material to a biologically active amendment. When appropriately optimized, thermal conversion enables the valorization of citrus waste into a biochar with potential agronomic benefits. These findings emphasize the necessity of standardized phytotoxicity testing and careful selection of production parameters prior to agricultural application, particularly when biochar is intended to influence early stages of plant development.

## Figures and Tables

**Table 1:** Concentrations of hazardous elements determined by ICP-MS, values on dry basis. Values are means ± SE (n  =  3). Different letters (a–f) indicate significant differences based on Tukey’s test (*p* < 0.05).

Sample	Cr	Ni	Cu	Zn	As	Cd	Pb	Hg
	mg·kg^−1^	mg·kg^−1^	mg·kg^−1^	mg·kg^−1^	mg·kg^−1^	mg·kg^−1^	mg·kg^−1^	mg·kg^−1^
Orange peel	2.51± 0.08 a	3.95 ± 0.31 a	2.71 ± 0.03 a	9.79 ± 0.32 a	0.11 ± 0.01 a	<0.02	0.09 ± 0.01 a	0.0051 ± 0.00 a
250 °C	5.81 ± 0.07 b	6.53 ± 0.03 b	4.46 ± 0.04 b	19.56 ± 1.84 b	0.73 ± 0.02 b	<0.02	0.14 ± 0.00 b	0.0013 ±0.00 a
300 °C	6.78 ± 0.65 bc	7.69 ± 0.57 c	5.36 ± 0.07 c	26.18 ± 5.57 b	0.84 ± 0.00 b	<0.02	0.17 ± 0.00 b	0.0033 ± 0.00 b
350 °C	6.24 ± 0.36 c	8.56 ± 0.64 cd	6.08 ± 0.06 d	18.11 ± 0.61 bc	0.94 ± 0,.07 bc	<0.02	0.26 ± 0.01 c	0.0008 ± 0.00 c
450 °C	7.02 ± 1.19 c	9.56 ± 1.20 d	8.22 ± 0.29 e	22.19 ± 0.18 c	0.83 ± 0.02 bc	<0.02	0.25 ± 0.02 c	0.0008 ± 0.00 c
550 °C	7.41 ± 0.16 c	9.62 ± 0.11 d	6.89 ± 0.10 f	17.33 ± 0.40 d	0.76 ± 0.04 c	<0.02	0.22 ± 0.01 c	0.0039 ±0.00 d

**Table 2:** The pH and electrical conductivity of aqueous extracts.

Sample	pH	Electrical Conductivity (μS·cm^−1^)
Orange peel	4.26	1861
250 °C	6.53	1936
300 °C	7.56	771.8
350 °C	9.5	884.2
450 °C	10.01	2259
550 °C	10.06	3080

**Table 3:** Total phenolics (TPC), flavonoids (TFC), and total antioxidant activity (TAA) of the methanol extract from orange peel. Values are means ± SE (n  =  3). Different letters (a–g) indicate significant differences based on Tukey’s test (*p* < 0.05). GAE—equivalents of gallic acid; equivalents of quercetin; equivalents of ascorbic acid.

Sample	TPC (GAE mg·g^−1^ DW)	TFC (QE mg·g^−1^ DW)	TAA (AAE mg·g^−1^ DW)
Orange peel	53.84 ± 0.69 d	90.05 ± 3.4 c	27.58 ± 0.05 g
250 °C	4.77 ± 0.08 c	8.07 ± 0.04 b	6.32 ± 0.09 f
300 °C	2.22 ± 0.03 b	3.52 ± 0.05 ab	2.87 ± 0.02 e
350 °C	0.57 ± 0.01 a	1.34 ± 0.02 a	1.12 ± 0.04 d
450 °C	0.24 ± 0.01 a	1.17 ± 0.07 a	0.64 ± 0.06 c
550 °C	0.05 ± 0.01 a	0.32 ± 0.01 a	0.39 ± 0.02 b

**Table 4:** Characteristics related to seed germination, seedling vigor, and root/shoot (R/S) ratio of durum wheat (*Triticum durum* Desf.) and common buckwheat (*Fagopyrum esculentum* Moench.) as affected by aqueous extracts of orange peel biochar at different concentrations. Statistical evaluation was performed using Tukey’s HSD test (*p* < 0.05); significant differences among treatments are indicated by different letters. GIe—Germination index of aqueous extracts.

Plant	Temperature of Pyrolysis (°C)	GIe (%)	Germination on the 3rd Day (%)	Germination on the 6th Day (%)	Germination Rate (%)	Germination Index (-)	Seedling Vigor Index I. (mm)	Seedling Vigor Index II. (mg)	Seedling Vigor Index III. (mg)	R/S_Length 3rd Day (-)	R/S_Length 6th Day (-)	R/S_Fresh_Weight (-)	R/S_Dry_Weight (-)
		Mean ± SE	Mean ± SE	Mean ± SE	Mean ± SE	Mean ± SE	Mean ± SE	Mean ± SE	Mean ± SE	Mean ± SE	Mean ± SE	Mean ± SE	Mean ± SE
***Triticum durum***	Control	100 ± 4.69 d	96.67 ± 1.05 bc	97.33 ± 0.67 bc	99.31 ± 0.69 bc	54.21 ± 3.98 d	121.26 ± 7.19 def	2.91 ± 0.06 b	0.21 ± 0.01 a	1.82 ± 0.04 cde	1.86 ± 0.05 f	1.29 ± 0.12 h	0.73 ± 0.15 b
	No treatment	-	-	-	-	-	-	-	-	-	-	-	-
	250	54 ± 4.13 b	92 ± 2.26 b	96.67 ± 1.83 bc	95.24 ± 2.3 b	47.25 ± 1.18 bcd	78.48 ± 1.81 b	4.7 ± 0.16 d	0.27 ± 0 01 a	1.60 ± 0.02 bcde	0.76 ± 0.03 bc	1.10 ± 0.02 fgh	0.93 ± 0.07 b
	300	81 ± 5.61 bcd	95.33 ± 1.33 bc	98.67 ± 0.82 bc	96.64 ± 1.49 bc	54.24 ± 2.52 bcd	85.93 ± 2.54 bc	4.19 ± 0.19 cd	0.24 ± 0.02 a	1.73 ± 0.06 cde	0.63 ± 0.02 b	1.11 ±0.09 gf	0.94 ± 0.17 b
	350	67 ± 2.7 bc	95.33 ± 1.49 b	98 ± 1.33 bc	95.29 ± 1.71 b	53.23 ± 0.66 bcd	115.06 ± 4.86 cde	4.52 ± 0.39 d	0.34 ± 0.04 a	1.42 ± 0.05 bcd	0.90 ± 0.08 bcd	0.94 ± 0.05 efg	1.05 ± 0.1 b
	450	75 ± 5.99 bcd	96.67 ± 1.05 bc	98.67 ± 0.82 bc	97.98 ± 0.83 bc	52.05 ± 0.86 cd	137.04 ± 5.15 efg	5.03 ± 0.21 d	0.44 ± 0.02 a	1.29 ± 0.12 bc	1.10 ± 0.11 cde	0.76 ± 0.02 bdef	0.77 ± 0.02 b
	550	94 ± 7.15 cd	100 ± 0 c	100 ± 0 c	100 ± 0 c	51.10 ± 1.28 bcd	144.03 ± 4.74 efgh	4.95 ± 0.19 d	0.41 ± 0.02 a	1.37 ± 0.04 bc	1.03 ± 0.07 bcd	0.65 ± 0.02 bcde	0.70 ± 0.04 b
***Fagopyrum esculentum***	Control	100 ± 3.89 d	96 ± 1.94 bc	96 ± 1.94 bc	100 ± 0 c	46.66 ± 1.69 bcd	91.75 ± 4.83 bc	2.58 ± 0.22 b	0.24 ± 0.02 a	2.29 ± 0.45 e	1.46 ± 0.19 f	0.36 ± 0.02 bc	0.18 ± 0.01 a
	No treatment	-	-	-	-	-	-	-	-	-	-	-	-
	250	104 ± 3.02 d	94.67 ± 1.33 bc	94.67 ± 1.33 b	100 ± 0 c	43.78 ± 0.99 b	103.29 ± 4.41 bcd	2.17 ± 0.25 b	0.21 ± 0.01 a	1.73 ± 0.12 cde	1.19 ± 0.08 de	0.27 ± 0.06 ab	0.25 ± 0.02 a
	300	138 ± 12.59 e	100 ± 0 c	100 ± 0 c	100 ± 0 c	47.10 ± 0.83 bcd	166.95 ± 8.22 h	2.34 ± 0.31 b	1.43 ± 1.18 a	0.80 ± 0.09 ab	1.00 ± 0.1 bcd	0.46 ± 0.15 bcd	0.17 ± 0.04 a
	350	177 ± 11.68 fg	100 ± 0 c	100 ± 0 c	100 ± 0 c	46.80 ± 0.51 bcd	128.41 ± 8.87 defg	3.07 ± 0.16 bc	0.24 ± 0.01 a	2.23 ± 0.35 de	1.12 ± 0.12 cde	0.32 ± 0.03 aabc	0.22 ± 0.02 a
	450	206 ± 8.48 g	96.67 ± 1.05 bc	96.67 ± 1.05 bc	100 ± 0 c	48.05 ± 1.3 bcd	149.34 ± 11.9 fgh	3.10 ± 0.52 bc	0.64 ± 0.39 a	1.27 ± 0.12 bc	0.99 ± 0.1 bcd	0.36 ± 0.12 bc	0.18 ± 0.05 a
	550	155 ± 4.92 ef	100 ± 0 c	100 ± 0 c	100 ± 0 c	45.50 ± 1.08 bc	157.51 ± 5.02 gh	4.10 ± 0.09 cd	0.25 ± 0.01 a	1.32 ± 0.05 cde	1.31 ± 0.07 de	0.23 ± 0.03 ab	0.11 ± 0.02 a

**Table 5:** Characteristics related to seed germination, seedling vigor, and root/shoot (R/S) ratio of durum wheat (*Triticum durum* Desf.) and common buckwheat (*Fagopyrum esculentum* Moench.) after treatment with aqueous extracts of orange peel biochar at different concentrations. Statistical analysis was performed using Tukey’s HSD test (*p* < 0.05); significant differences among treatments are indicated by different letters.

Plant	Temperature of Pyrolysis (°C)	Length of Root on the 3rd Day (mm)	Length of Root on the 6th Day (mm)	Length of Shoot on the 3rd Day (mm)	Length of Shoot on the 6th Day (mm)	Length of Seedling (mm)	Weight of Fresh Root (g)	Weight of Fresh Shoot (g)	Weight of Fresh Seedling (g)	Weight of Dried Root (g)	Weight of Dried Shoot (g)	Weight of Dried Seedling (g)
		Mean ± SE	Mean ± SE	Mean ± SE	Mean ± SE	Mean ± SE	Mean ± SE	Mean ± SE	Mean ± SE	Mean ± SE	Mean ± SE	Mean ± SE
***Triticum durum***	Control	54.46 ± 2.23 fg	94.41 ± 6.03 cde	30.01 ± 1.51 def	51.35 ± 4.63 b	145.76 ± 10.62 b	1.66 ± 0.06 c	1.33 ± 0.1 b	2.99 ± 0.07 bc	0.09 ± 0.02 bc	0.13 ± 0.01 a	0.21 ± 0.01 a
	No treatment	-	-		-	-	-	-	-	-	-	-
	250	30.87 ± 1.58 b	61.88 ± 1.18 b	19.31 ± 0.99 bcd	81.81 ± 3.99 cde	143.69 ± 4.83 b	2.55 ± 0.08 e	2.32 ± 0.09 cde	4.86 ± 0.17 e	0.14 ± 0.01 de	0.15 ± 0.01 a	0.29 ± 0.02 a
	300	44.37 ± 2.53 cdef	61.41 ± 2.58 b	25.65 ± 1.15 cdef	97.23 ± 2.43 def	158.64 ± 4.21 bc	2.24 ± 0.18 de	2.01 ± 0.06 bcd	4.25 ± 0.2 de	0.11 ± 0.01 cd	0.13 ± 0.01 a	0.24 ± 0.02 a
	350	37.85 ± 1.39 bc	90.73 ± 4.37 cd	26.62 ± 0.39 cdef	101.73 ± 4.71 def	92.45 ± 4.37 cd	2.23 ± 0.22 de	2.36 ± 0.14 cde	4.59 ± 0.36 e	0.17 ± 0.02 ef	0.17 ± 0.02 a	0.34 ± 0.04 a
	450	40.87 ± 3.25 bcde	106.36 ± 4.34 def	32.45 ± 3.16 ef	99.06 ± 8.14 def	205.41 ± 9.48 ef	2.2 ± 0.1 de	2.9 ± 0.11 def	5.1 ± 0.2 e	0.19 ± 0.01 f	0.25 ± 0.01 a	0.44 ± 0.02 a
	550	49.32 ± 3.76 def	107.93 ± 6.36 def	36.1 ± 3.08 f	104.85 ± 3.31 ef	212.78 ± 6.93 ef	1.96 ± 0.1 cd	2.99 ± 0.09 ef	4.95 ± 0.19 e	0.17 ± 0.01 ef	0.24 ± 0.01 a	0.41 ± 0.02 a
***Fagopyrum esculentum***	Control	30.22 ± 0.72 b	80.73 ± 2.84 b	14.68 ± 1.97 b	58.41 ± 5.78 b	139.15 ± 4.62 b	0.7 ± 0.05 b	1.98 ± 0.15 bc	2.67 ± 0.19 b	0.04 ± 0 ab	0.21 ± 0.02 a	0.25 ± 0.02 a
	No treatment	-	-	-	-	-	-	-	-	-	-	-
	250	31.91 ± 0.75 b	90.29 ± 3.72 cd	18.95 ± 1.75 bc	76.56 ± 4.37 bcd	66.85 ± 5.94 bc	0.49 ± 0.12 ab	1.81 ± 0.19 bc	2.3 ± 0.27 b	0.04 ± 0 ab	0.17 ± 0.01 a	0.22 ± 0.01 a
	300	39.92 ± 3.65 bcd	116.25 ± 6.56 ef	50.7 ± 2.9 g	118.7 ± 8.98 f	234.95 ± 10.88 f	0.62 ± 0.03 b	1.73 ± 0.3 bc	2.34 ± 0.31 b	0.05 ± 0 ab	1.38 ± 1.18 a	1.43 ± 1.18 a
	350	51.48 ± 3.39 efg	103.59 ± 6.53 cde	24.82 ± 2.78 bcde	93.50 ± 7.44 def	97.09 ± 12.81 d	0.75 ± 0.07 b	2.32 ± 0.12 cde	3.07 ± 0.16 bcd	0.04 ± 0 ab	0.2 ± 0.01 a	0.24 ± 0.01 a
	450	61.66 ± 1.94 g	104.21 ± 7.63 def	50.05 ± 4.06 g	106.86 ± 4.78 ef	211.07 ± 6.96 ef	0.67 ± 0.04 b	2.52 ± 0.49 cdef	3.19 ± 0.52 bcd	0.04 ± 0 ab	0.63 ± 0.42 a	0.67 ± 0.42 a
	550	44.9 ± 1.43 cdef	123.32 ± 3.18 f	34.19 ± 2.03 ef	95.38 ± 5.03 def	218.7 ± 6.55 ef	0.77 ± 0.08 b	3.33 ± 0.06 f	4.1 ± 0.09 cde	0.03 ± 0.01 a	0.23 ± 0.01 a	0.25 ± 0.01 a

## Data Availability

All the data are included in the main text.
